# Phylogeny, biogeography and methodology: a meta-analytic perspective on heterogeneity in adult marine turtle survival rates

**DOI:** 10.1038/s41598-018-24262-w

**Published:** 2018-04-11

**Authors:** Joseph B. Pfaller, Milani Chaloupka, Alan B. Bolten, Karen A. Bjorndal

**Affiliations:** 1 Caretta Research Project, Savannah, GA USA; 2 Archie Carr Center for Sea Turtle Research and Department of Biology, University of Florida, Gainesville, FL USA; 3 Ecological Modelling Services Pty Ltd, University of Queensland, St. Lucia, Queensland Australia

## Abstract

Comparative syntheses of key demographic parameters are critical not only for identifying data gaps, but also for evaluating sources of heterogeneity among estimates. Because demographic studies frequently exhibit heterogeneity, evaluating sources of heterogeneity among estimates can inform biological patterns and conservation actions more broadly. To better understand adult survival in marine turtles and avoid drawing inaccurate conclusions from current estimates, we conducted a comprehensive meta-analysis to test how heterogeneity among estimates was partitioned among phylogenetic, biogeographic and methodological factors. Fifty-nine studies from five marine turtle species met the minimum selection criteria for inclusion in our meta-analysis. Heterogeneity among survival estimates was first partitioned between differences in ocean basin (Indo-Pacific *versus* Atlantic), then by differences among family/tribe within the Indo-Pacific (Chelonini *versus* Carettini and Dermochelidae). However, apparent differences attributed to biogeography (ocean basin effect) and phylogeny (family/tribe effect) were highly correlated with methodological differences in tag type, model type, habitat type and study duration, thereby confounding biological interpretations and complicating efforts to use many current survival estimates in population assessments. Our results highlight the importance of evaluating sources of heterogeneity when interpreting patterns among similar demographic studies and directly inform efforts to identify research priorities for marine turtles globally.

## Introduction

Demographic studies in ecology and conservation biology form the basis for assessing population viability and managing ecological risk^1^. However, estimates of key demographic parameters, such as survival and recruitment, viewed in isolation often provide limited and/or potentially biased inferences. In this regard, comparative syntheses of similar demographic studies are critical not only for identifying data gaps, but also for evaluating sources of heterogeneity among estimates. Because demographic studies frequently exhibit heterogeneity due to system-specific nature of biological phenomena^2^ and study-specific differences in methodology, identifying important sources of heterogeneity can inform biological patterns and conservation actions more broadly^2–4^. Conversely, comparative studies that fail to evaluate sources of heterogeneity risk drawing inaccurate conclusions and misleading management decisions.

We conducted a systematic review and comprehensive meta-analysis^5,6^ of annual survival rates for adult marine turtles to generate precision-weighted, species-specific estimates and prediction intervals from existing data^7^ and to explicitly model sources of heterogeneity among estimates^2^. In theory, because all marine turtles exhibit conserved life history patterns, including slow growth and delayed sexual maturity^8^, adult survival rates should be high (>0.90) and exhibit limited natural heterogeneity among estimates. Our goal was to quantify heterogeneity among survival estimates and test how heterogeneity is influenced by (a) phylogeny, (b) biogeography and (c) methodology. If heterogeneity is influenced by phylogeny or biogeography, then biological differences in survival among species/populations may exist. In this case, survival estimates that are lower than expected would highlight important species- or region-specific hotspots in adult mortality – areas of conservation concern. However, if heterogeneity is also strongly influenced by methodological differences, then statistical biases related to certain methodologies introduce artificial variation and mask important biological differences. In this case, the use of survival estimates that are lower than expected may mislead management decisions if methodological biases are not accounted for. We expect the findings of this synthesis to highlight the importance of evaluating sources of heterogeneity when interpreting demographic estimates^2^ and directly inform efforts to identify research priorities for marine turtles globally^9–12^.

## Methods

### Literature review and selection criteria

We followed the PRISMA protocols for assembling a dataset suitable for meta-analytic evaluation^5,6^. Specifically, we conducted a two-tiered literature search to compile annual survival probability estimates for adult marine turtles. A structured search was conducted in Google Scholar, Sea Turtle Document Library (seaturtle.org) and Sea Turtle Online Bibliography (Archie Carr Center for Sea Turtle Research, University of Florida) using the following Boolean search terms: survival, survivorship, mortality, and the names of the seven marine turtle genera. Then, an unstructured literature search was conducted by reviewing the reference lists of all the relevant publications and reports from the structured search. References compiled from the structured and unstructured search were reviewed and only those that estimated annual survival probabilities for adult turtles were retained (i.e., multiyear or stage-based estimates and estimates for juveniles were excluded). Seventy-eight survival estimates were found in the global literature review with 59 meeting the minimum selection criteria for inclusion in the meta-analysis (Supplementary Table S1). Marine turtle taxonomy/phylogeny follows Duchene *et al*.^13^. Dermochelidae (leatherbacks) is sister to Chelonidae, which includes Chelonini (flatback and green turtles) and Carettini (hawksbill, loggerhead and ridley turtles). The measure of effect size and precision of each estimate was the study-specific inverse-precision weighted annual survival probability and the associated standard error, respectively.

### Informative covariates

The following potentially informative covariates (or moderators) were compiled for all survival estimates: specific publication ID, research group (based on common authors), type of publication (journal or report), publication year, taxonomic group (species, tribe and family), study site, geographic region with subgrouping for two oceans (Indo-Pacific and Atlantic), study duration (years), habitat type (nesting or foraging), tag type and statistical estimation type with subgrouping for modeling procedures versus enumeration calculations. Each estimate was categorized by whether tag loss, imperfect detection and temporary emigration were explicitly accounted for or not. The population of interest in each study was also characterized in terms of population size, population trend, direct harvest history and fisheries bycatch impact using primarily Wallace *et al*.^14,15^. Details on covariate characterization can be found in the Supplementary Methods. Study duration and publication year were also used to account for various forms of publication bias^16,17^.

### Statistical modeling approach

We used a precision-weighted random-effects model approach to summarize the 59 survival estimates into species-specific subgroups before accounting for informative covariates^17–19^. In this 2-level analysis, the random effect was the specific study. Each study-specific survival estimate was a response variable soft-bound between 0–1. Response variables were therefore logit-transformed prior to fitting the random-effects models to ensure the predicted estimates were also between 0–1^20^ and then back-transformed for any predicted summaries. Species-specific random-effects models were fitted using the *metafor* package for R^21^ and displayed in a subgroup forest plot that was augmented with the species-specific random effects estimates and the prediction intervals for those estimates^7^.

Because many of the potentially informative covariates in our meta-analysis were highly correlated, we could not isolate individual effects or explicitly model interactions among covariates simultaneously. Therefore, we first used a recursive partitioning or conditional inference regression tree approach^22,23^ to explore underlying patterns among the potential effects of 12 correlated covariates on the 59 survival estimates. The conditional inference tree model was fitted and then displayed using the *partykit* package for R^24^ with a minimum split criterion of 0.95 (so P-value < 0.05). Because the conditional inference tree approach does not account for the precision of survival estimates, we also tested a series of meta-regression models using *metafor* to explore other potentially informative covariates when estimate precision was explicitly considered. Models with different predictors were compared based on Akaike Information Criterion (AICc).

We then used a mixed-effects meta-regression approach^19,25^ to model annual survival rates conditioned on the main interaction effect – ‘ocean *by* family/tribe’ – identified within the conditional inference tree and four additional moderators identified by exploratory meta-regression modeling – tag type, model type, habitat type and study duration. We also included a 3-level hierarchical structure^26,27^ to test for potential non-independence between studies conducted by the same research group (based on common authors), an important assumption to account for in meta-analytic studies^17,28^. In this 3-level hierarchical analysis, the random effects were study within research group. The mixed effects meta-regression models were fitted using a multivariate parameterization to accommodate assessment of random effect structures^29^. The model-predicted study-specific inverse-precision weighted annual survival estimates and covariate effects were summarized as covariate-specific boxplots. Continuous variables, like study duration, were modeled using B-splines (*splines* package)^30^ to account for potential nonlinear functional forms^29^ and predicted estimates were summarized using a cubic regression spline GAM-based smoother^31^ implemented within the *ggplots2* package for R^32^.

The I^2^ statistic^2,33^ was used to assess the level of unexplained heterogeneity estimated in the 3-level hierarchical meta-regression model fit to the 59 studies, and a simple R^2^ measure appropriate for the multivariate parameterization of a meta-regression model was used as a metric of the overall model fit. The Cochrane Q_E_ test was used as a formal test of residual heterogeneity^34^, while an omnibus *F*-test was used to test for significance of the set of all covariates included in the meta-regression model^35^. Lastly, we explored potential publication bias^16,17^ using a contour-enhanced funnel plot^36^ of the model predicted study-specific survival estimates using *metafor*.

### Data availability

The dataset supporting this article has been uploaded as part of the electronic supplementary material.

## Results

### Study- and species-specific survival estimates

**Figure 1:**
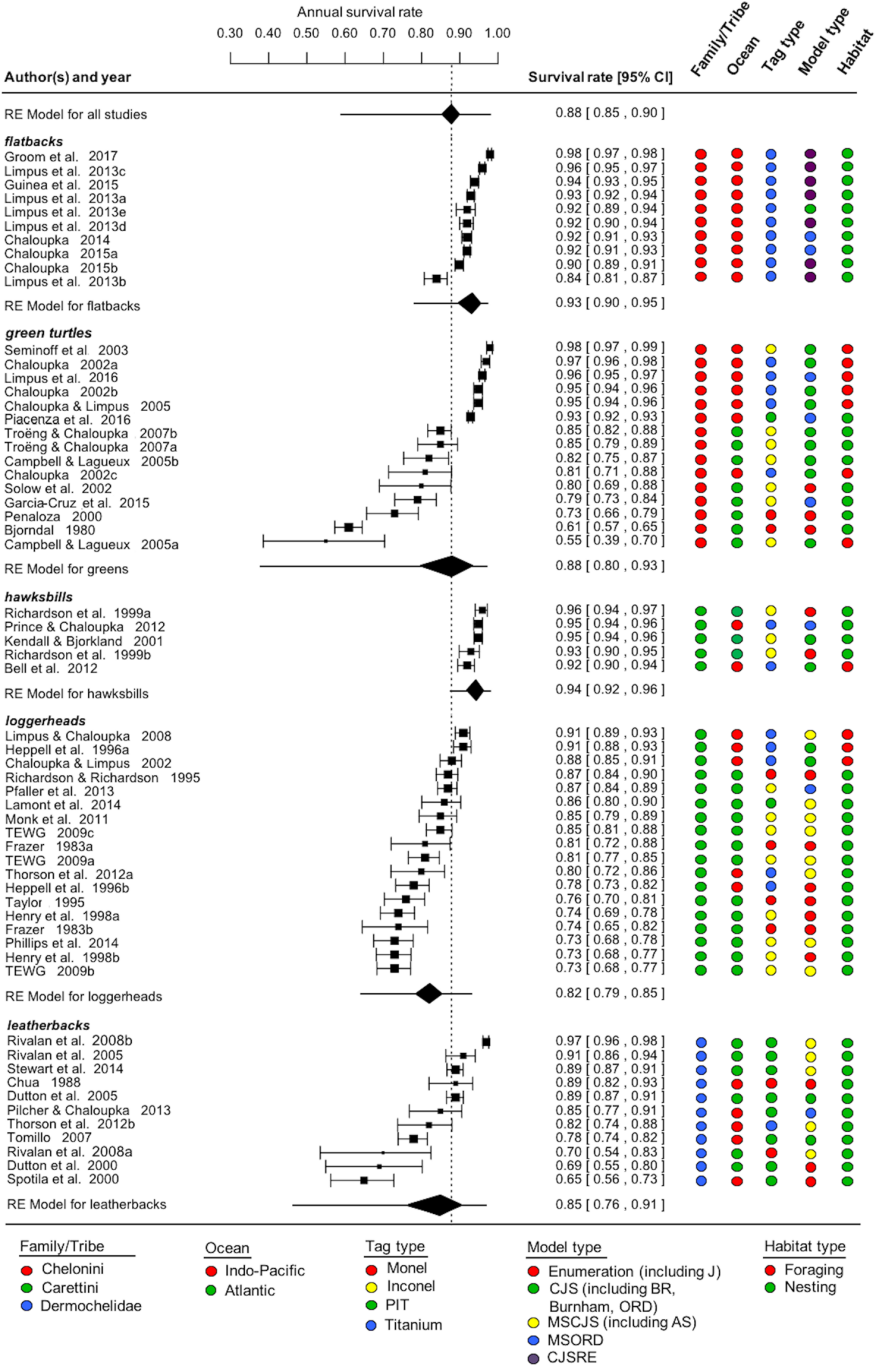
Random-effects forest plot of the inverse-variance weighted annual survival estimates for the 59 marine turtle studies (letters after reference year indicate different estimates from the same reference)^37^,^40^–^42^,^51^–^88^. The species-specific pooled or random-effect survival rate estimates (RE diamonds) are shown in addition to the prediction intervals (horizontal bar through each RE diamond). Plot ordered by effect size within each species and solid square = survival rate and size of symbol reflects relative weighting, horizontal bars = 95% confidence interval of each study-specific survival rate. Colored icons show potentially informative study-specific covariates.

The 59 species-specific survival rate estimates derived from the random-effects model are summarized in Fig. 1. Here the random-effect was the specific study. It is apparent that there was considerable species-specific heterogeneity in adult survival rate estimates. This was especially apparent for green and leatherback turtles that might be attributable to geographic and methodological factors. This apparent heterogeneity was explored further using hierarchical or multilevel meta-regression modeling to account for potentially informative phylogenetic^4^, geographic and methodological covariates. We have also included the species-specific prediction intervals in the subgroup forest plot, which shows for instance that a new flatback adult survival rate study would be expected to provide an annual estimate ca. 0.93 (95% prediction interval: 0.77–0.98). On the other hand, for leatherbacks it would be 0.85 (95% prediction interval: 0.45–0.97).

### Informative covariates

**Figure 2:**
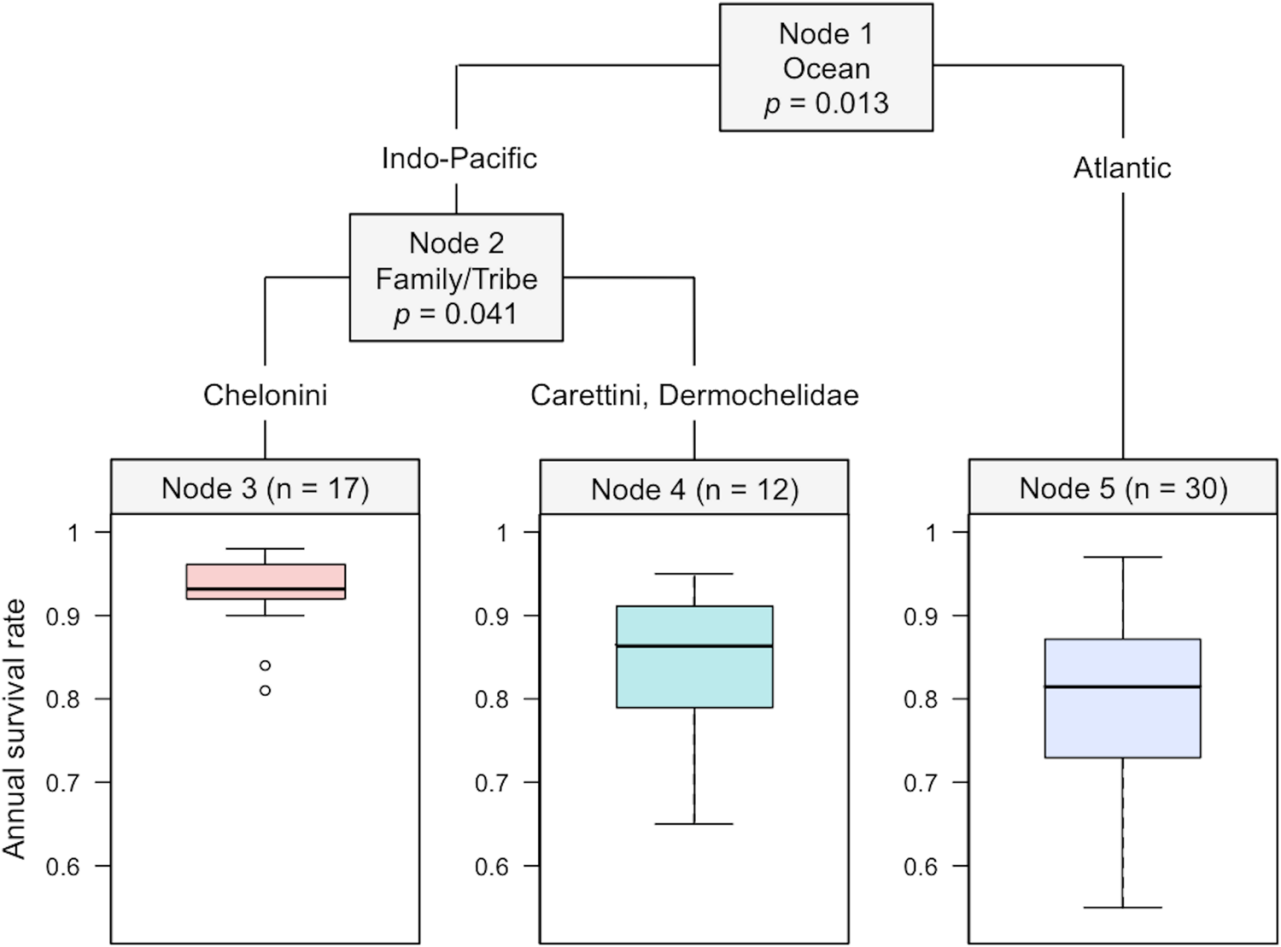
Conditional inference tree visualization of the effect of potentially informative covariates on the study-specific marine turtle survival rates.

The conditional inference tree approach identified two significant nodes at a minimum split criterion of 0.95: (Node 1) Atlantic versus Indo-Pacific oceans and (Node 2) Chelonini versus Carettini and Dermochelidae nested within the Indo-Pacific Ocean (Fig. 2). No other covariates remained in the final tree model at 0.95. The three terminal nodes are boxplot summaries of the model-derived survival estimates (n = sample size within a terminal node). In addition to ocean and species, the best-fitting models from the exploratory meta-regression approach (estimates weighted by precision) included tag type (and tag loss), model type, habitat type and study duration, suggesting the methodology as well as phylogeny and biogeography might have important effects on adult marine turtle survival rates.

### Covariate-specific model-predicted survival estimates

The 3-level hierarchical meta-regression model (random effects = study within research group) was a better fit than a 2-level model (random effect = study) (log-likelihood ratio test = 58.1, df = 2, P < 0.001). The 3-level model with a nonlinear functional form for study duration was a better fit than the same model with a linear functional form (log-likelihood ratio test = 19.7, df = 2, P < 0.001). The inclusion of the six moderators in the accepted 3-level, nonlinear regression model led to a significant improvement in overall model fit (Q_E_ = 121.4, df = 14, P < 0.001). However, ca. 97% of unaccounted variance was due to residual heterogeneity (tau^2^ = 0.005, 95% CI: 0.003–0.011; I^2^ = 97.2), and only ca. 37% of the residual heterogeneity in the accepted model was accounted for by inclusion of the six moderators (R^2^ = 0.37). This high level of residual heterogeneity was significant (Q_E_ = 508.6, df = 44, P < 0.001) and apparently typical of ecological studies^2,28^.

**Figure 3:**
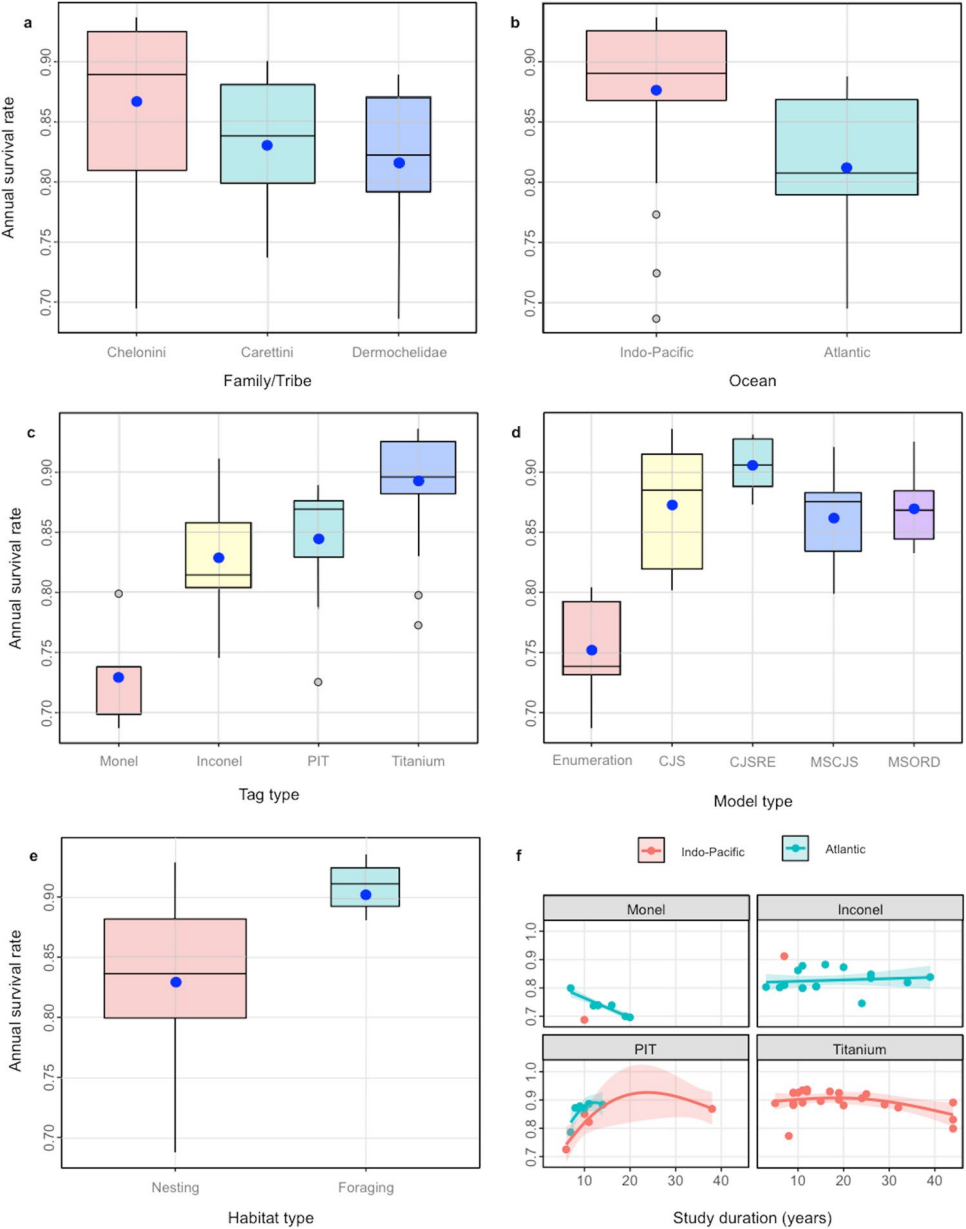
Multi-panel display summarizing the covariate-specific model-predicted estimates derived from the 3-level hierarchical meta-regression model fit to the 59 studies conditioned on the following covariates: (**a**) ocean (2 levels), (**b**) family (3 levels), (**c**) tag type (4 levels), (**d**) model type (5 levels), (**e**) habitat type (2 levels) and (**f**) study-duration by tag-type interaction term. Closed blue dots in the boxplots shows the predicted mean, horizontal bar = predicted median. The underlying trend in the tag-specific study-duration subplots (bottom right panel) shown by a cubic regression spline smooth with an inverse-precision weighted 95% confidence polygon.

**Figure 4:**
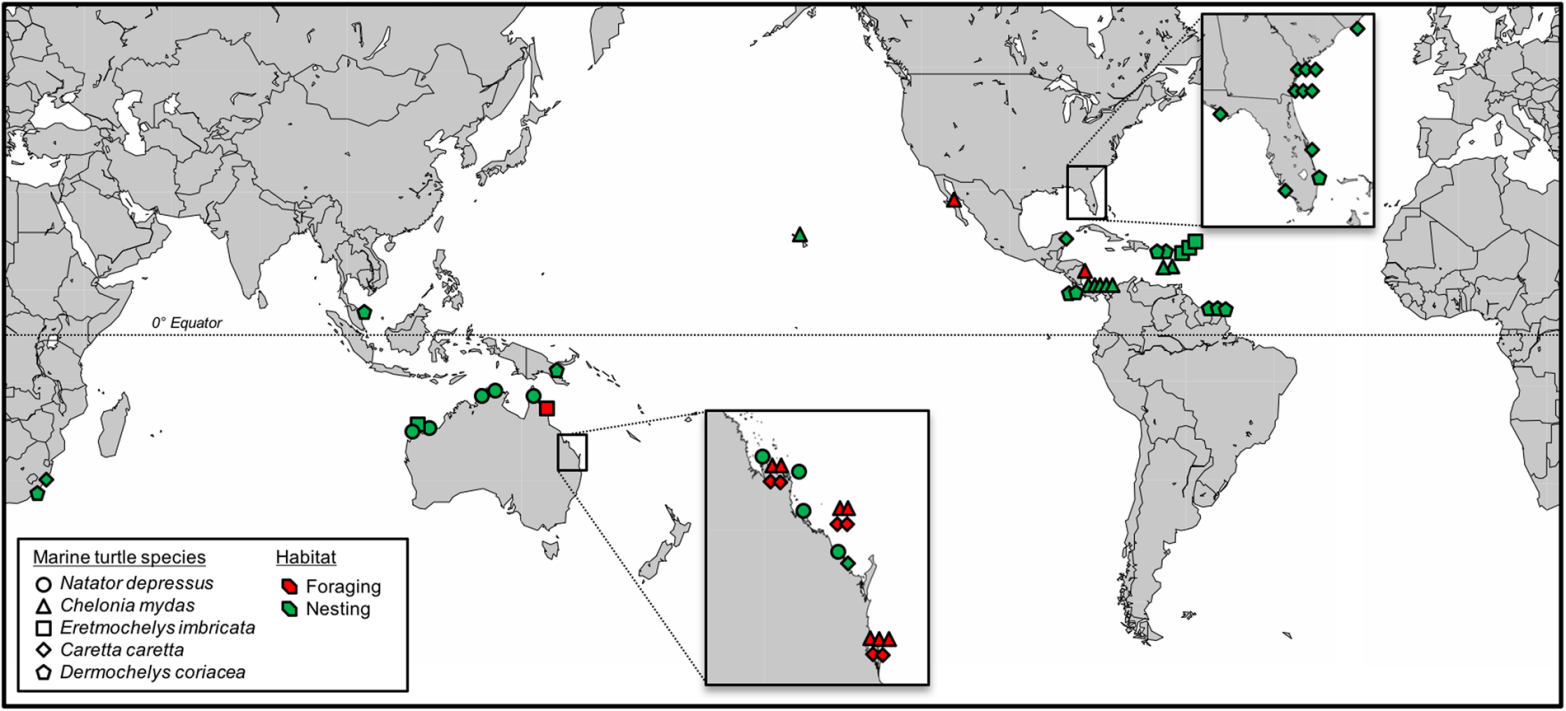
Global geographic distribution of the 59 survival estimates included in the meta-analysis. Icon shapes indicate different marine turtle species and colors indicate different habitats (foraging grounds *versus* nesting beaches). The map was generated using the Maptool function at www.seaturtle.org^89^.

Covariate-specific survival estimates were derived from the 3-level hierarchical meta-regression model fit to the 59 studies conditioned on family/tribe (3 levels), ocean (2 levels), tag type (4 levels), method type (5 levels), habitat type (2 levels) and the study duration by tag type interaction term (Fig. 3). General patterns among model-predicted survival estimates include: (1) Chelonini > Carettini ~ Dermochelidae (Fig. 3a), (2) Indo-Pacific > Atlantic (Fig. 3b), (3) titanium > inconel ~ PIT > monel (Fig. 3c), (4) statistical modeling > enumeration (Fig. 3d) and (5) foraging areas > nesting beaches (Fig. 3e). While tag type was mostly ocean-specific, study duration appears to have no effect or a negative effect on survival estimates from metal tags and a positive effect on survival estimates from PIT tags, though most are of relatively short duration (Fig. 3f).

### Publication bias

A contour-enhanced funnel plot of the predicted study-specific survival estimates derived from the 3-level hierarchical meta-regression model fit to the 59 studies showed no evidence of any form of publication bias (Supplementary Fig. S1). There was also no temporal effect of publication year in the conditional inference tree or the exploratory meta-regression approach. Therefore, we found no evidence of publication bias in the marine turtle survival rates for which we could test for using a range of approaches.

## Discussion

Annual survival estimates for adult marine turtles exhibit considerable heterogeneity. Survival estimates were generated from five turtle species, three major oceans and five decades of research involving numerous methodological techniques. For this reason, heterogeneity among estimates might be expected. However, because adult survival is a key demographic parameter and marine turtles exhibit conserved life-history patterns, including slow growth and delayed sexual maturity^8^, the extent of heterogeneity found among survival estimates complicates accurate biological interpretations. To better understand heterogeneity in adult marine turtle survival rates, we conducted a comprehensive meta-analysis to test how heterogeneity among estimates was partitioned among phylogenetic, biogeographic and methodological factors. Results from this study represent an important step towards identifying the key factors that drive differences among survival estimates and setting research and management priorities for marine turtles globally^11,12^.

### Evaluating sources of heterogeneity

Results from the recursive partitioning or conditional inference regression tree approach^22,23^ indicate that heterogeneity among survival estimates was first partitioned between differences in ocean basin (Indo-Pacific *versus* Atlantic), then by differences among family/tribe within the Indo-Pacific (Chelonini *versus* Carettini and Dermochelidae). Though often ignored, phylogenetic non-independence can change the results of ecological meta-analyses^4^. However, in this case, differences were not associated with phylogenetic similarity – Carettini is more closely related to Chelonini (both in Chelonidae) than to Dermochelidae^13^. Therefore, survival estimates in Carettini and Dermochelidae are likely similar and lower than Chelonini due to other biological or methodological factors, not phylogenetic history. Estimates for Indo-Pacific Chelonini might be higher because this group includes flatback turtles, which are endemic to the Indo-Pacific, exhibit different and potentially less vulnerable habitat-use patterns (exclusively neritic versus both neritic and oceanic), and have been consistently monitored with robust methodologies (see below)^37^. Estimates specifically from the Northwest Atlantic (as there are no estimates from the Northeast or South Atlantic) are lower than the Indo-Pacific. However, this is not consistent with any apparent differences in natural or anthropogenic mortality rates or any known differences in life-history behavior among populations in different oceans. Predictors that might be indicative of differences in adult mortality among turtle populations such as harvest history and fisheries bycatch impact^14,15^ were not found to contribute to heterogeneity among survival estimates (Fig. 2). Therefore, our results suggest that while heterogeneity is connected to biogeography (ocean effect) and phylogeny (family/tribe effect), differences cannot be attributed to phylogenetic similarity or apparent regional differences in adult mortality. Instead, region- and species-specific differences appear to be tightly linked to the use of different methodologies applied in different oceans and therefore to a different composition of turtle species/populations.

The ocean and family/tribe predictors were highly correlated with methodological predictors, including tag type, method/model type, habitat type and study duration. Results from exploratory meta-regression modeling showed that these four factors, in addition to ocean and species, were consistently found within the best-fitting models. Direct monitoring of marine turtles in the wild is inherently difficult and CMR studies remain the most reliable tool for estimating annual survival rates. However, such efforts are clearly vulnerable to statistical biases when methodologies cannot account for complexities inherent to the biology and study of marine turtles such as imperfect detection, tag loss, and temporary and permanent emigration. First, our results highlight the importance of tag type and its ramifications on tag loss on survival estimates. We found that estimates generated from monel tags were consistently lower than estimates generated primarily from inconel, titanium and PIT tags. Estimates generated from monel tags are likely biased low and effected by study duration (Fig. 3f) due to factors related to higher rates of tag loss and subsequent individual misidentification: monel tags have lower retention rates^38^, tend to corrode in seawater^39^ and were often applied only singly in early studies (e.g.^40,41^). Similar concerns with respect to tag loss may apply to inconel tags, although to a lesser extent than monel. Second, our results show that early attempts to estimate survival via enumeration methods that rely on time-series counts of individuals violate far more CMR assumptions than statistical modeling procedures such as CJSRE and MSORD, which are designed to estimate and account for biases including imperfect detection and temporary emigration^42,43^. Although robust statistical modeling procedures are well accepted and almost universally used, the accuracy of estimates will always depend on the quality of CMR data to which the models are applied. Third, our results suggest that CMR data collected in foraging areas may be less prone to statistical biases than data collected on nesting beaches. Because marine turtles spend considerably more time in foraging areas, estimates from foraging areas may be more robust to statistical biases associated with temporary, as well as permanent, emigration. Methodological differences clearly contribute to heterogeneity among survival estimates.

Patterns among different methodologies and their associated biases are highly correlated with biological patterns: more low estimates come from the Atlantic Ocean, Carettini and Dermochelidae, monel tags, enumeration calculations and nesting beaches, while more high estimates come from the Indo-Pacific ocean, Chelonini, titanium tags, statistical modeling procedures and foraging areas (Figs 1 and 4). We proposed that if heterogeneity was influenced by phylogeny or biogeography, then biological differences in adult survival may exist and management action should be directed towards species/populations associated with survival estimates that are relatively low. However, this interpretation was contingent on whether or not heterogeneity was also influenced by methodology. Because heterogeneity among survival estimates was strongly influenced by different methodologies that were highly correlated with phylogenetic and biogeographic differences, we cannot make accurate biological interpretations to confidently direct management actions. Whether estimates that are lower than expected indicate elevated rates of adult mortality or simply highlight the use less robust methodologies is currently undecipherable. For this reason, efforts to apply many current survival estimates to model population viability and interpret long-term trends risk drawing inaccurate conclusions and misleading management actions. This highlights the need to critically evaluate current survival estimates and attempt to correct or account for statistical biases when possible. Additionally, future estimates should strive to apply robust methodologies that eliminate or minimize statistical biases, so that the detection of important region- or species-specific differences in adult mortality are not masked by heterogeneity associated with differences in methodology. Because adult mortality from over-harvesting and fisheries bycatch remains a significant conservation concern for marine turtles^14,15^, more work is needed to generate new, more comparable estimates that will improve the accuracy of assessments of the status and trends in marine turtle populations^9,10,44^ and help guide conservation measures and management in the future.

### Identifying data gaps

Our systematic review of annual survival estimates for adult marine turtles revealed important region- and species-specific data gaps that should be high priorities for research to support conservation^11,12^. At the region-level for all applicable species, there is a noticeable lack of estimates from the eastern North Atlantic (including the Mediterranean Sea), South Atlantic, western and northern Indian Ocean (including the Red Sea and Persian Gulf), western North Pacific and eastern South Pacific (Fig. 4), all areas that support globally important marine turtle populations^14^ and some that also host long-term research and monitoring projects capable of generating estimates of adult survival rates. At the species-level, we found estimates for all seven marine turtle species. However, all estimates for Kemp’s and olive ridley turtles (n = 4 and 1, respectively) were excluded for not meeting the minimum selection criteria for inclusion in the meta-analysis. Because these estimates were not deemed sufficiently robust and all are at least two decades old, new estimates for ridleys should be a research priority, especially given their susceptibility to overexploitation from human threats such as fisheries bycatch^45^. Globally, estimates from nesting beaches are far more frequent than estimates from foraging habitats, which are primarily from eastern Australia (Fig. 4). Although logistically more challenging, priority should be given to estimating survival rates in foraging habitats, especially in the well-studied northwest Atlantic where estimates from nesting beaches overwhelmingly predominate.

Survival estimates from unstudied regions and species will not only improve assessments of specific populations, but will also improve our understanding of the important factors that affect survival rates and allow us to better decipher biological versus methodological sources of heterogeneity among estimates. New estimates generated from traditional CMR data should avoid methodologies that may generate artificially low estimates of survival rates if possible and, if used, should acknowledge and attempt to account for statistical biases associated with those methods. Future studies may strive to implement standardized robust methodologies that eliminate or minimize potential biases and isolate the biological signal resulting from actual sources of mortality. Though not yet practically implementable in most cases, the standardized use of population-wide genetic “tagging” applied in both nesting and foraging areas holds great potential for future studies. Estimates using this approach generated across many regions and species would be directly comparable, allowing researchers and managers to identify regional hotspots in marine turtle mortality and implement more effective management strategies at a global scale.

### The value of meta-analysis

Meta-analysis is a powerful tool ideally suited for combining the results of demographic studies. Because demographic studies in ecology and conservation biology often exhibit considerable heterogeneity^2^, estimates of key parameters, such as adult survival rates, viewed in isolation may provide limited and/or potentially biased inferences. Our results show the value of meta-analysis in generating robust species-specific demographic estimates and identifying data gaps, but also highlight the importance of evaluating sources of heterogeneity when interpreting patterns among similar demographic studies. Because demographic studies frequently exhibit heterogeneity due to system-specific nature of biological phenomena^2^ and study-specific differences in methodology, identifying important sources of heterogeneity can inform biological patterns and conservation actions more broadly^2–4^. Synthesized data are essential for modelling wildlife population dynamics exposed to various anthropogenic hazards^46^ and for testing ecological hypotheses such as the importance or not of dispersal for species exposed to habitat loss^47^. Because there are currently few meta-analyses of wildlife demographic rates^3,48^, this approach represents a productive and important area for future research.

Global research on marine turtles is poised to benefit from the application of meta-analysis. The accumulation of demographic and biological information from decades of research across all species and biogeographic regions has elicited global assessments and numerous review articles in recent years (e.g.^14,15,44,49,50^). Meta-analytic comparisons of key parameters and threats associated with the biology and conservation of marine turtles − such as breeding and recruitment rates, population and individual growth rates, and fisheries bycatch and plastic ingestion rates − will reveal novel patterns and valuable insights, while accounting for important sources of heterogeneity that may obscure important interpretations^2–4,25,28^. Such analyses serve to guide research and conservation priorities for marine turtles into the future.

## Electronic supplementary material

Supplementary materials

Table S1
