# Supplementary material for: Phylogeny, biogeography and methodology: a meta-analytic perspective on heterogeneity in adult marine turtle survival rates
Source: Sci Rep. 2018 Apr 11;8:5852. doi: 10.1038/s41598-018-24262-w (PMC5895625; doi:10.1038/s41598-018-24262-w)
Supplement: Supplementary file 1 — Supplementary materials [file 41598_2018_24262_MOESM1_ESM.doc]

Electronic Supplementary Material

Phylogeny, biogeography and methodology: a meta-analytic perspective on heterogeneity in adult marine turtle survival rates

Joseph B. Pfaller*, Milani Chaloupka, Alan B. Bolten & Karen A. Bjorndal

*Corresponding author

Supplementary methods

Selection criteria

All estimates that were included in the final meta-analysis were required to meet several criteria. First, estimates from the same study must apply sufficiently different methods or models for multiple estimates to be included. Second, estimates from grey literature must represent the only source for a given estimate, i.e., if two estimates were the same (or very similar) in both grey literature sources and published articles (i.e., same authors, time series and method), then only the published article was retained. Third, estimates must be accompanied with a measurement of standard error (se) or with sufficient statistical information for a measurement of standard error to be calculated [e.g., lower and upper 95% confidence intervals (lci and uci), sample size (n), or t-statistic]. If standard error was not presented but sufficient information was provided, then standard error was calculated in one of four ways: (A) from 95% confidence intervals [se = (uci-lci)/(2*1.96)], (B) from linear regression analysis [se = estimate/t-statistic)], (C) from sample size [se = sqrt((estimate*(1-estimate))/n)], or (D) from annual estimates by averaging s, uci and lci for all years and using method (B) to estimate se from confidence intervals. Last, estimates must be generated using field methodologies or statistical procedures that are comparable among studies (i.e., primarily CMR studies) and meet a minimum standard of plausibility (i.e., >0.5).

Covariates or predictors

The 25 predictors that were in considered in the analyses were:

1. Estimate ID: unique modifier for each estimate, labeled as an in-text citation.
2. Study ID: unique modifier for each study (i.e., some studies have more than one estimate).
3. Publication type: (i) journal or (ii) report (includes abstracts and theses).
4. Publication year: year of study publication or presentation.
5. Research group: based on commonalities among listed authors.
6. Ocean: (i) northwest Atlantic, (ii) northeast Pacific, (iii) central Pacific, (iv) southwest Pacific, (v) northern Australia, (vi) southeast Indian or (vii) southwest Indian. (ii-vii) were also grouped into a 2-ocean “Indo-Pacific” subgroup.
7. Study site: name of specific location
8. Habitat: (i) nesting or (ii) foraging
9. Turtle species: (i) loggerhead (*Caretta caretta*), (ii) green turtle (*Chelonia mydas*), (iii) flatback (*Natator depressus*), (iv) hawksbill (*Eretmochelys imbricata*), or (v) leatherback (*Dermochelys coriacea*).
10. Taxonomy: (i) Chelonini (green and flatback turtles), (ii) Carettini (loggerhead, hawksbill and ridley turtles), or Dermochelidae (leatherback turtles).
11. Population size: (i) small (<700 annual nesting females), (ii) medium (700-7000 annual nesting females), or (iii) large >7000 annual nesting females) based on data compiled by Wallace et al. (2011).
12. Population trend: (i) increasing, (ii) stable or (iii) decreasing based on long-term trends in the number of annual nesting females from data primarily compiled by Wallace et al. (2011).
13. Harvest history: (i) yes or (ii) no.
14. Bycatch impact: (i) high or (ii) low based on classifications in Table 6 of Wallace et al. (2013).
15. Start year: first year in the study data series.
16. Duration: the number of years in the study data series.
17. Data: (i) CMR or (ii) not CMR.
18. Method: (i) enumeration or (ii) model.
19. Model: (i) other (for enumeration studies), (ii) Arnason-Schwarz (AS), (iii) band recovery (BR), (iv) Burnham, (v) Cormack-Jolly-Seber (CJS), (vi) Cormack-Jolly-Seber CJSre); (vii) Jolly, (viii) open robust design (ORD), (ix) multistate Cormack-Jolly-Seber (MSCJS), or (x) multistate open robust design (MSORD).
20. Detection bias: (i) explicitly modeled or accounted for bias; (ii) not explicitly accounted for, but diagnostic tests show no need to explicitly account for bias; (iii) not explicitly accounted for or addressed with diagnostic tests.
21. Temporary emigration bias: same options as (19)
22. Permanent emigration (or transience) bias: same options as (19)
23. Tag loss bias: same options as (19)
24. Tag type: (i) monel only, (ii) primarily inconel, (iii) primarily titanium or (iv) primarily PIT (passive integrated transponder).
25. Standard error calculation: (i) explicitly calculated in study, (ii) calculated using method (A) from above, (iii) calculated using method (B) from above, (iv) calculated with method (C) from above, or (v) calculated using method (D) from above.

Supplementary Results

A total of 78 annual survival estimates for adult marine turtles was found in the literature review (Table S1) and 59 met the selection criteria for inclusion in the final meta-analysis. Nine estimates were excluded for not presenting a measurement of standard error or sufficient statistical information for a measurement of standard error to be calculated. Five estimates were excluded for using field methodologies or statistical procedures that were not comparable to other studies or for not meeting a minimum standard of plausibility (<0.50). Two estimates were excluded because the same (or a very similar) estimate was found in a published article. All estimates for Kemps ridley (N = 4) and olive ridley (N = 1) turtles were excluded for one of the following reasons.

Supplementary references (not included in main text)

Dutton, D. L., Dutton, P. H., Boulon, R. Coles, W. C. & Chaloupka, M. New insights into population biology of leatherbacks from 20 years of research: profile of a Caribbean population in recovery. *NOAA Tech Memo NMFS-SEFSC-503* (2003).

Garcia-Cruz, M., Lampo, M., Penaloza, C., Sole, G. & Rodriquez-Clark, K. Population trends and survivorship of nesting green sea turtles on Isla de Aves, Venezuela. *NOAA Tech Memo NMFS-SEFSC-645* (2013).

Garcia-Cruz, M. A. Demografía, genética y epibiontes de la población de tortuga verde (*Chelonia mydas*) que se reproduce en Isla de Aves, Venezuela . *Instituto Venezolano de Investigaciones Científicas* (2014).

Hedges, M. E. & Berkson, J. An estimation of demographic parameters for the loggerhead sea turtle, *Caretta caretta*, on Bald Head Island, NC USA. *Twenty sixth annual symposium on sea turtle biology and conservation. International Sea Turtle Society* (2006).

Heppell, S. S., *et al.* A population model to estimate recovery time, population size, and management impacts on Kemp's ridley sea turtles. *Chelonian Conservation and Biology* **4,** 767-773 (2005).

Kendall, W. L., *et al.* Comparison of the demography of two Caribbean nesting populations of hawksbill turtle. *NOAA Tech Memo NMFS-SEFSC-631* (2012).

Marquez, R. & Doi, T. A trial of theoretical analysis on population of Pacific green sea turtle, *Chelonia mydas carrinegra* Caldwell, in waters of Gulf of California, Mexico. *Bull. Toka. Fish. Res. Lab.***73** (1973).

Marquez, R., Villaneuva, O. A. & Sanchez Perez, M. The population of the Kemp's ridley sea turtle in the Gulf of Mexico - *Lepidochelys kempii* in *Biology and conservation of sea turtles* (ed Bjorndal, K. A.) 159-164 (Smithsonian Institution Press, 1982).

Marquez, R., Penaflores, S. C., Villanueva, O. A. & Diaz, J. F. A model for diagnosis of populations of olive ridleys and green turtles of West Pacific tropical coasts in *Biology and conservation of sea turtles* (ed. Bjorndal, K. A.) 153-158 (Smithsonian Institution Press, 1982).

NMFS (National Marine Fisheries Service). Stock assessment of loggerhead and leatherback sea turtles and an assessment of the impact of the pelagic longline fishery on the loggerhead and leatherback sea turtles of the western North Atlantic. *NOAA-Tech Memo NMFS-SEFSC-445* (2001).

Parmenter, C. J. & Limpus, C. J. Female recruitment, reproductive longevity and inferred hatchling survivorship for the flatback turtle (*Natator depressus*) at a major eastern Australian rookery. *Copeia* **2,** 474-477 (1995).

Sarti, L., Eckert, S. A., Ninel Garcia, T. & Barragan, A. R. Decline of the world's largest nesting assemblage of leatherback turtles. *Marine Turtle Newsletter* **74,** 2-5 (1996).

Sasso, C. R., Epperly, S. P. & Johnson, C. Annual survival of loggerhead sea turtles (*Caretta caretta*) nesting in pennisular Florida: a cause for concern. *Herptol. Conservation Biol.***6,** 443-448 (2011).

TEWG (Turtle Expert Working Group). An assessment of the Kemp's ridley (*Lepidochelys kempii*) and loggerhead (*Caretta caretta*) sea turtle populations in the western north Atlantic. *NOAA Tech Memo NMFS-SEFSC-409* (1998).

Supplementary tables

**Supplementary Table S1.** Seventy-eight annual survival estimates from adult marine turtles collated from a two-tiered literature search and associated meta-data for each estimate (see accompanying Excel file).

Supplementary figures

**Supplementary Figure S1.** Contour-enhanced funnel plot of model-predicted estimates of the study-specific survival rates derived from the 3-level hierarchical meta-regression model fit to the 59 studies. There was no evidence of funnel plot asymmetry.
